# Urine metabolic phenotyping in children with nocturnal enuresis and comorbid neurobehavioral disorders

**DOI:** 10.1038/s41598-021-96104-1

**Published:** 2021-08-16

**Authors:** Mei-Ching Yu, Ta-Min Wang, Yee-Hsuan Chiou, Meng-Kung Yu, Chiao-Fan Lin, Chih-Yung Chiu

**Affiliations:** 1 Division of Pediatric Nephrology, Department of Pediatrics, Lin-Kou Chang Gung Memorial Hospital and College of Medicine, Chang Gung University, 5, Fusing Street, Gueishan, Taoyuan 333 Taiwan; 2 Division of Pediatric Urology, Department of Urology, Lin-Kou Chang Gung Memorial Hospital and College of Medicine, Chang Gung University, Taoyuan, Taiwan; 3 Department of Pediatrics, Kaohsiung Veterans General Hospital, Kaohsiung, Taiwan; 4 Department of Pediatrics, Taichung Tzu Chi Hospital, Buddhist Tzu Chi Medical Foundation, Taichung, Taiwan; 5 Department of Child and Adolescent Psychiatry, Lin-Kou Chang Gung Memorial Hospital, Taoyuan, Taiwan; 6 Division of Pediatric Pulmonology, Department of Pediatrics, Clinical Metabolomics Core Laboratory, Lin-Kou Chang Gung Memorial Hospital and College of Medicine, Chang Gung University, 5, Fusing Street, Gueishan, Taoyuan 333 Taiwan

**Keywords:** Biomarkers, Diseases, Medical research, Urology

## Abstract

Nocturnal enuresis (NE) is a common problem among 10% school-aged children. The etiologies underlying childhood NE is complex and not fully understood nowadays. Nevertheless, increasing evidence suggests a potential link between neurobehavioral disorders and enuresis in children. In this study, we aimed to explore novel metabolomic insights into the pathophysiology of NE and also, its association with pediatric psychiatric problems. Urine collected from 41 bedwetting children and 27 healthy control children was analyzed by using ^1^H-nuclear magnetic resonance spectroscopy from August 2017 to December 2018. At regular follow-up, there were 14 children with refractory NE having a diagnosis of attention deficient hyperactivity disorder (ADHD) or anxiety. Eventually, we identified eight significantly differential urinary metabolites and particularly increased urinary excretion of betaine, creatine and guanidinoacetate linked to glycine, serine and threonine metabolism were associated with a comorbidity of neurobehavioral disorders in refractory bedwetting children. Notably, based on physiological functions of betaine acting as a renal osmolyte and methyl group donor, we speculated its potential role in modulation of renal and/or central circadian clock systems, becoming a useful urinary metabolic marker in diagnosis of treatment-resistant NE in children affected by these two disorders.

## Introduction

Nocturnal enuresis (NE), also known as bedwetting, is defined as involuntary urination while asleep in children above 5 years of age by the International Children’s Continence Society^1^. Based on the association with daytime voiding dysfunction, it can be further categorized into mono-symptomatic nocturnal enuresis (MNE) and non-monosymptomatic nocturnal enuresis (NMNE), respectively^2^. The literatures reported the prevalence of childhood NE is highly variable among different countries, ranging from 2.3 to 25%^3–5^. In Taiwan, there was 6.8% of elementary-school children reported to have the bedwetting problem^5^. Overall, the global prevalence of NE is around 10% among school-aged children, particularly predominance in boys^6^. Notably, if bedwetting children and adolescents are not treated properly or ignored without treatment, there is 2% of the population persistent into adulthood^7^.

Until the present, the exact pathophysiology of childhood NE remains unclarified and is likely to be multifactorial, involving biological, developmental, genetic, psychosocial, and environmental aspects^8^. Theoretically, nocturnal polyuria caused by vasopressin deficiency, detrusor overactivity and high arousal thresholds have been regarded as the three major pathogenetic mechanisms for MNE. In this context, pharmacological therapy consisting of desmopressin, anticholinergics (e.g., oxybutynin and tolterodine), or tricyclic antidepressants (e.g., imipramine, amitriptyline and desipramine) and bedwetting alarms become the mainstay of clinical management and treatment in the present days^1^. However, 40% of bedwetting children and adolescents still have insufficient responses to the standard treatment or experience a relapse after stopping treatment, particularly for those with NMNE^1,9^. This reflects beyond the three-system model, there are other factors involved in enuresis pathogenesis.

In the early era, bedwetting was viewed as a primarily pediatric psychiatric disorder. Since the end of twentieth century, however, the bio-behavioral perspective on childhood NE has considerably grown and indicates enuresis is more likely the result of developmental delays than psychiatric illness^8^. Nevertheless, numerous clinical studies and population-based studies have uncovered the strong association between enuresis and attention deficit hyperactivity disorder (ADHD)^10–15^. In this modern world, ADHD is the most common neurodevelopmental disorder affecting 7.2–15.5% of school-aged children and adolescents, particularly in boys^16^. Furthermore, it is evident that ADHD children are vulnerable to NE and other voiding disturbances, such as daytime incontinence, urgency and frequency^16^. The prevalence estimates of enuresis in children with ADHD have been reported in 22–32%^12,13^. On the other hand, 30–40% of bedwetting children ages six to 12 years had a diagnosis of different types of ADHD^11,14^. Although psychiatric problems and NE are still a “chicken-and-egg” situation, this bidirectional relationship between the two disorders is potentially explained by deficits in arousal and/or developmental delays in central nervous system^17,18^.

Among contemporary research, metabolomic approaches have been widely applied in diverse clinical conditions, allowing assessment of metabolic pathways and networks liked with genetic and environmental factors. In this study, we attempted to characterize urine metabolic phenotyping in relation to childhood NE using ^1^H-nuclear magnetic resonance (NMR) spectroscopy. Furthermore, we also aimed to explore potentially useful metabolic markers and relevant pathways associated with a comorbidity of neurobehavioral disorders such as ADHD in children particularly with treatment-resistant NE.

## Results

### Participant characteristics

A total of 68 participants consisting of 41 children with NE (age: 8.9 ± 2.4 years; male/female:22/19) and 27 healthy controls (age:7.4 ± 0.5 years; male/female:10/17) were enrolled in this study. During 3- to 32-month follow-up of drug treatment, 14 bedwetting children had a diagnosis of neurobehavioral or emotional disorders (age: 8.9 ± 2.5 years; male/female: 7/7), including 10 cases of ADHD, two cases of ADHD and coexisting autism spectrum disorder, and two cases of anxiety disorder, based on the Diagnostic and Statistical Manual of Mental Disorders, fourth edition^19^. Of those 14 children with both NE and neuropsychiatric problems, there were 12 cases of MNE and two cases of NMNE. Table 1 showed demographic data of all participants. The baseline characteristics (i.e., gender, height, weight and body mass index) were no different between the three groups, bedwetting children with and without ADHD or anxiety, and health controls. Regarding frequent or severe bedwetting (> three episodes per week), there was male predominance of 61.9% (13/21) in the subgroup of childhood NE without ADHD or anxiety. On the other hand, males and females with childhood NE were at equal risk for a comorbidity of ADHD or anxiety. During the follow-up period, more than half of children with both NE and neurobehavioral disorders (57%) were resistant to the combination medications of Minirin® and oxybutynin or imipramine, whereas 52% of the bedwetting children without comorbid ADHD or anxiety had adequate response to the monotherapy with Minirin®.

**Table 1:** The basic demographic data of study subjects.

Variables	Childhood NE (N = 41)		Healthy controls (N = 27)	*P* value ^ϕ^
	ADHD/anxiety group, N = 14 (%)	Non-ADHD/anxiety group, N = 27 (%)		
Age (years)	8.9 ± 2.5	8.9 ± 2.3	7.4 ± 0.5	0.09
Gender, male(M): female(F)	7:7	15:12	10:17	0.38
Height (cm)	126.5 ± 15.6	129.0 ± 15.3	125.0 ± 6.91	0.59
Weight (kg)	28.7 ± 8.6	30.5 ± 10.2	24.8 ± 4.7	0.17
BMI (kg/m^2^)	17.7 ± 3.1	18.1 ± 4.0	15.8 ± 2.1	0.05
**Frequency of NE (per week)**				
< 3 episodes	2(14%) M: F = 1:1	6 (22%) M: F = 2:4		
3–6 episodes	8(57%) M: F = 4:4	17 (63%) M: F = 9:8		
7 episodes	4(29%) M: F = 2:2	4 (15%) M: F = 4:0		
Daytime voiding dysfunction	2 (14%)	1 (4%)		
**Medications (during follow-up)**				
DDAVP	5 (36%)	14(52%)		
Combined drugs	8 (57%)	9(33%)		
No drugs	1 (7%)	4 (15%)		

*Means ± standard deviation is given for each variable and each group.

^ψ^The statistics is analyzed by Kruskai–Wallis test and *p* < 0.05 is statistically significant (GraphPad Prism, version 8.4.2.).

### Urinary metabolic phenotyping differentiates children with NE and the population subgroups of the affected children with/without concomitant neurobehavioral disorders from health controls

**Figure 1:**
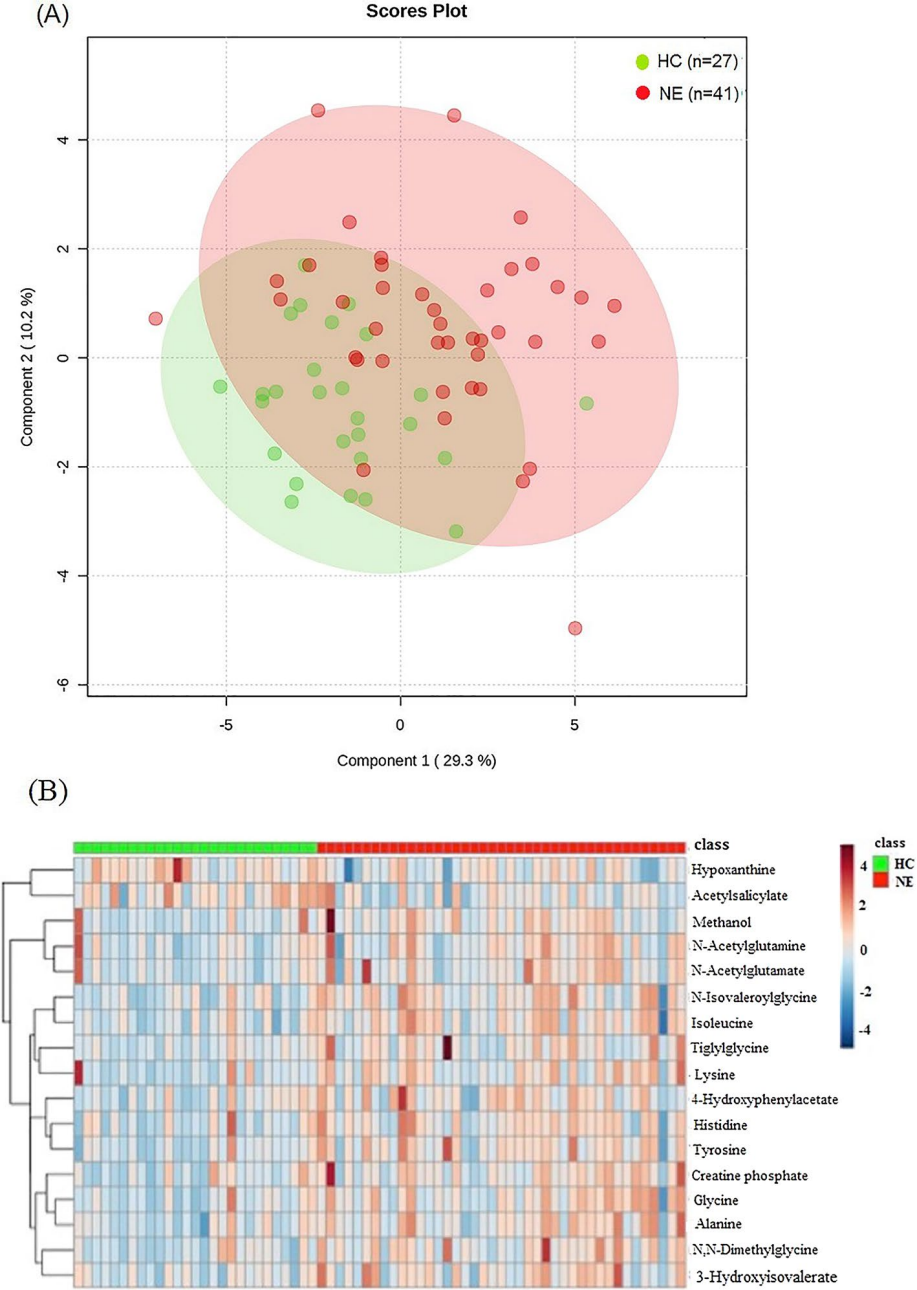
(**A**) The PLS-DA of 1H NMR urine spectra is measured from 41 children with NE (red colored dots) and 27 healthy controls (green colored dots). The score plot shows the model discrimination between the two groups based on the global metabolites identified in urine. 29.3% and 10.2% are the scores of components 1 and 2, respectively. (**B**) In the heat map, it illustrates the degree of the 17 differential urinary metabolites discovered contributes the group separation.

In Fig. 1A, PLS-DA illustrated the recognition of the two groups, children with NE and healthy controls, according to global metabolites discovered in urine. Based on the VIP score (≥ 1.0) and fold change of the identified metabolic species, 17 urinary metabolites significantly differentially expressed in the group of childhood NE compared with healthy controls (*p* < 0.05), shown in Table 2. These 17 characteristic urinary metabolites were N-isovaleroylglycine, isoleucine, glycine, alanine, N, N-dimethylglycine (DMG), tyrosine, creatine phosphate, lysine, histidine, 3-hydroxyisovalerate, tiglylglycine, N-acetylglutamate, 4-hydroxyphenylacetate, methanol, hypoxanthine, N-acetylglutamine and acetylsalicylate. Accordingly, a clustering analysis of the differential urinary metabolites was illustrated in the heatmap (Fig. 1B), displaying the degree of individual metabolite of interest contributed to the group distinction.

**Table 2:** The results from VIP score and fold change (FC) of urinary metabolites significantly differentially expressed between bedwetting children with (n = 14) and without (n = 27) comorbid ADHD or anxiety disorder, and healthy controls (n = 27) (*p* < *0.05*).

Metabolite	NE versus HC			NE with ADHD/anxiety versus HC			NE without ADHD/anxiety versus HC			NE with ADHD/anxiety versus NE without ADHD/anxiety		
	VIP score	FC	*P value*	VIP score	FC	*P value*	VIP score	FC	*P value*	VIP score	FC	*P value*
N-Isovaleroylglycine	2.09	1.83	**0.001**	1.74	2.10	**0.004**	2.21	1.69	**0.002**	0.73	1.24	0.487
Isoleucine	2.02	1.76	**0.001**	1.66	1.93	**0.006**	2.14	1.67	**0.004**	0.63	1.16	0.548
Glycine	1.82	1.55	**0.001**	1.62	1.74	**0.003**	1.82	1.45	**0.007**	0.90	1.20	0.341
Alanine	1.69	1.50	**0.001**	1.59	1.69	**0.001**	1.62	1.40	**0.010**	1.01	1.20	0.248
N, N-Dimethylglycine	1.58	1.42	**0.001**	0.97	1.26	**0.021**	1.92	1.50	**0.002**	0.65	0.84	0.423
Tyrosine	1.55	1.38	**0.001**	1.31	1.43	**0.002**	1.63	1.36	**0.004**	0.42	1.06	0.591
Creatine phosphate	1.23	1.28	**0.007**	1.26	1.50	**0.005**	1.09	1.17	**0.035**	1.16	1.28	0.122
Lysine	1.61	1.25	**0.007**	1.49	1.48	**0.012**	1.48	1.13	**0.041**	1.15	1.31	0.217
Histidine	1.10	1.20	**0.011**	0.94	1.23	**0.021**	1.10	1.19	**0.041**	0.40	1.04	0.567
3-Aminoisobutyrate	0.45	1.40	0.558	1.49	2.10	**0.036**	0.45	1.04	0.617	2.82	2.03	**0.026**
3-Hydroxyisovalerate	1.25	1.34	**0.009**	1.34	1.45	**0.001**	1.10	1.29	0.060	0.92	1.12	0.262
Tiglylglycine	1.42	1.70	**0.010**	1.78	1.82	**0.000**	1.11	1.63	0.097	1.48	1.12	0.128
N-Acetylglutamate	0.97	1.19	**0.016**	1.01	1.33	**0.011**	0.86	1.12	0.061	0.93	1.19	0.171
4-Hydroxyphenylacetate	1.13	1.29	**0.018**	1.11	1.33	**0.008**	1.07	1.28	0.072	0.55	1.04	0.499
Methanol	1.36	1.46	**0.022**	1.22	1.99	**0.042**	1.34	1.19	0.051	0.82	1.68	0.398
Leucine	0.75	1.30	0.240	1.38	1.48	**0.009**	0.26	1.21	0.747	1.72	1.23	0.107
Hypoxanthine	1.39	0.74	**0.006**	0.81	0.77	0.076	1.68	0.73	**0.008**	0.58	1.05	0.479
N-Acetylglutamine	0.71	1.11	**0.044**	0.44	1.10	0.202	0.87	1.11	**0.040**	0.16	0.99	0.788
Valine	0.74	1.24	0.160	1.40	1.48	**0.001**	0.20	1.11	0.752	1.85	1.33	**0.038**
Acetylsalicylate	1.42	0.70	**0.033**	1.00	0.72	0.117	1.58	0.69	0.055	0.03	1.04	0.981
trans-Aconitate	0.99	1.18	0.075	0.76	1.26	0.166	1.08	1.14	0.106	0.20	1.10	0.828
3-Hydroxyisobutyrate	1.05	1.44	0.055	1.17	1.65	**0.015**	0.92	1.33	0.155	0.84	1.24	0.385
Formate	0.89	1.50	0.176	1.19	1.54	**0.025**	0.68	1.48	0.412	0.90	1.04	0.438
Guanidoacetate	0.85	1.20	0.069	1.02	1.39	**0.028**	0.62	1.09	0.251	1.15	1.28	0.136
Betaine	1.21	1.40	0.051	1.32	1.81	**0.030**	0.98	1.18	0.187	1.30	1.53	0.198
Creatine	1.06	1.42	0.109	1.28	1.64	**0.036**	0.78	1.31	0.330	1.28	1.25	0.245
Taurine	0.95	1.37	0.065	1.01	1.70	**0.047**	0.88	1.19	0.110	0.87	1.43	0.336
N-Acetylcysteine	0.54	0.95	0.136	0.04	1.04	0.917	0.98	0.90	**0.014**	0.83	1.16	0.192
Dimethylamine	0.35	1.13	0.571	1.15	1.70	0.055	0.41	0.83	0.570	2.44	2.05	**0.010**

Furthermore, in comparison with non-bedwetting healthy children, the increased concentrations of 23 urinary metabolites were significant in children with NE and comorbid ADHD or anxiety disorder (*p* < 0.05) (Table 2). These 23 differentially expressed urinary metabolites were N-isovaleroylglycine, isoleucine, glycine, alanine, DMG, tyrosine, creatine phosphate, lysine, histidine, 3-aminoisobutyrate, 3-hydroxyisovalerate, tiglylglycine, N-acetylglutamate, 4-hydroxyphenylacetate, methanol, leucine, valine, 3-hydroxyisobutyrate, formate, guanidoacetate, betaine, creatine and taurine. Among these metabolites of interest, there were some overlapping metabolites of interest identified in the population subgroup of childhood NE but without ADHD (Table 2). Furthermore, there were three urinary metabolites, including valine, 3-aminoisobutyrate and dimethylamine, significantly higher expressed in children affected by the two disorders compared with bedwetting children without comorbid ADHD or anxiety (*p* < 0.05) (Table 2). All three are known physiological metabolites involved in brain functions and disease^20–22^.

### Identification of characteristic urinary metabolites and the relevant functional pathway in bedwetting children affected by the presence of ADHD or anxiety

**Figure 2:**
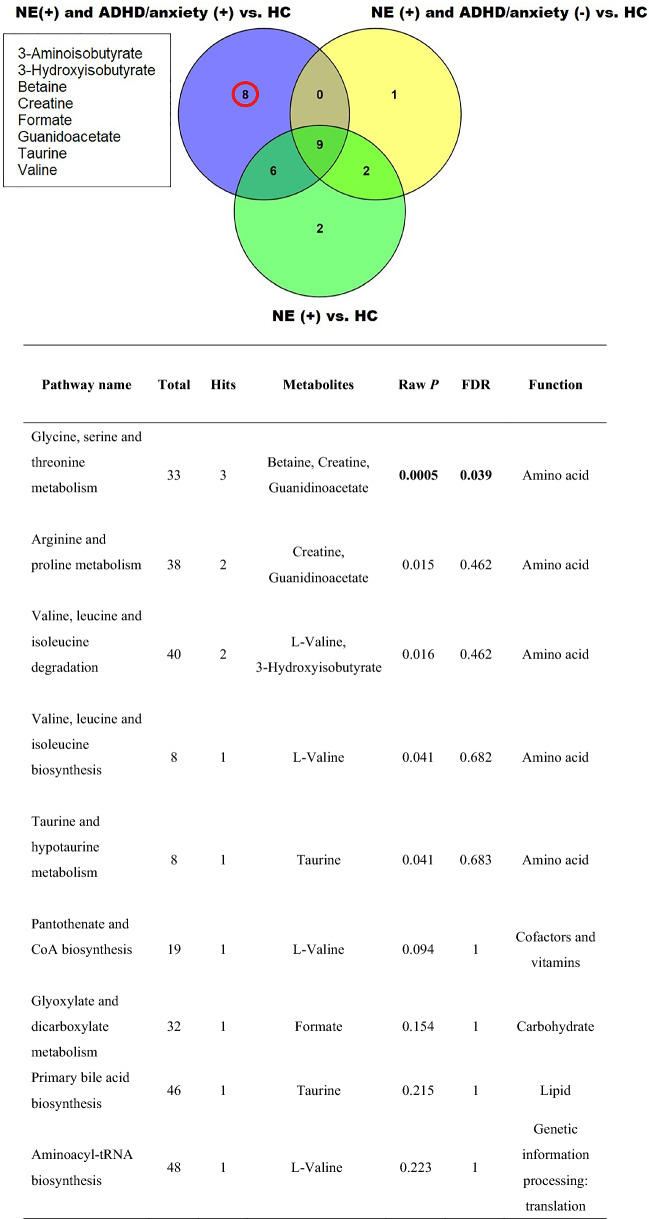
The three-cycle Venn diagram (the upper of the diagram) identifies several overlapping urinary metabolites from each data set, children with both NE and ADHD or anxiety versus health controls (HC), bedwetting children without ADHD or anxiety versus HC, and children with NE versus HC, respectively. As a result, nine urinary metabolites of interest are associated with childhood NE. Furthermore, eight characteristic urinary metabolites statistically link to co-occurrence of neurobehavioral problems in children with NE. The KEGG database is applied for functional metabolic pathway analysis (the bottom of the diagram), demonstrating urinary betaine, creatine and guanidinoacetate involved in glycine, serine and threonine metabolism (p = 0.0005 and FDR = 0.039) may have an influential role to play in children affected by these two disorders.

Among the three data sets consisting of NE vs. health controls, NE with comorbid ADHD or anxiety versus health controls, and NE without ADHD or anxiety vs. health controls, Venn diagram (Fig. 2) demonstrated the number of overlapping urinary metabolites from each data set. Apparently, there were nine characteristic urinary metabolites (i.e., alanine, creatine phosphate, glycine, histidine, isoleucine, lysine, DMG, N-isovaleroylglycine, and tyrosine) associated with childhood NE. Furthermore, eight characteristic urinary metabolites (i.e., 3-aminoisobutyrate, 3-hydroxyisobutyrate, betaine, creatine, formate, guanidoacetate, taurine and valine) linked to children with NE and comorbid ADHD or anxiety disorder. Tenfold internal cross-validation as well as permutation tests were performed to assess the quality of the resulting statistical models. Quality parameters Q2 (quality of prediction) and R2 (explained variance) were measured to judge the predictive power of PLS-DA among these three data sets (Supplemental Table S1). Subsequently, the KEGG pathway analysis revealed the changes of urinary betaine, creatine and guanidinoacetate concentrations involved in glycine, serine and threonine metabolism appeared to have statistically significant relations to a comorbidity of ADHD or anxiety in persistent bedwetting children. And, more than half of children affected by the two disorders were resistant to the standard medications and presented frequent NE (> three episodes per week).

## Discussion

In this study, ^1^H-NMR spectroscopy was applied to investigate global metabolomic profiling of urine from children with NE, particularly interest in biomarker discovery of children with both NE and neurobehavioral disorders. However, distinct clinical entities with overlapping manifestations, and untargeted metabolomics approach could be an obstacle to identification of specific metabolites. Nevertheless, through the integrated analysis of urine metabolomic data, the result uncovered the critical metabolic pathway involved in treatment- resistant NE in children affected by the presence of ADHD or anxiety. Also, several potential and characteristic urinary metabolic markers were identified in children affected by these two disorders, particularly urinary betaine is one of the most likely candidates in the pathogenesis.

Betaine (N, N, N-trimethylglycine) is a dietary nutrient essential for health. It is mostly obtained either from foods (e.g., wheat, spinach, beets and shellfish), or synthesized by mitochondrial oxidation of choline^23^. Betaine accumulates in many organs of the human body, including liver, kidneys and brain^24^. In the kidney, betaine is freely filtered through the glomerulus and nearly completely reabsorbed by the renal tubules. Physiologically, betaine functions as an organic osmolyte to protect cells against hyperosmotic stress (e.g., hypernatremia and hyperglycemia), and maintain cell volume without disrupting cell function. Besides, it is also a direct methyl-group donor via transmethylation for use in many biochemical pathways^25^. For example, betaine donates a methyl group to guanidinoacetate via methionine to produce creatine, which is beneficial for athletic performance and muscle strength^26^. Because of its dual roles, betaine deficiency or abnormalities in betaine metabolism is increasingly linked to various human diseases such as obesity, diabetes mellitus, cardiovascular and neuropsychiatric disorders^27–29^.

In the present study, we found children with NE and comorbid ADHD or anxiety presented excessive urinary betaine loss. Besides, increased urinary DMG excretion was also observed in bedwetting children compared with healthy controls. DMG is a metabolite of betaine, and normally excreted in urine or metabolized to sarcosine. It exerts an osmoregulatory influence on sodium excretion by the kidney, which is similar to betaine. Taken together, our result speculated the potential role of imbalances in renal osmolyte regulation in enuresis pathogenesis. Nocturnal polyuria caused by circadian rhythm disturbance of the antidiuretic pituitary hormone vasopressin has been considered as the important pathogenesis of childhood NE^30^. However, beyond the rhythm driven by hypothalamus (central clocks), many literatures reported disturbed renal circadian rhythm (local clocks) played an influential role in nocturnal polyuria^31,32^. For instance, increased nocturnal sodium excretion, disturbances in hormones responsive for water and sodium handling (e.g. angiotensin II, aldosterone and atrial natriuretic factor), higher urinary excretion of renal autacoid prostaglandin E2, higher nocturnal blood pressure and decreased glomerular filtration rate (GFR) overnight were reported^33,34^.

Recently, Gil and his colleagues applied NMR-based method to analyze urine samples from 277 patients with chronic kidney disease (CKD) at the stage 1–5^35^. They discovered several urinary metabolites significantly linked to severity and the progression of CKD. Particularly urinary betaine and myo-inositol concentrations were negatively correlated with annual declines in GFR. This finding was further validated by kidney transcriptome analysis, demonstrating decreased renal expressions of betaine and myo-inositol transporters in CKD mice. Besides, abnormally increased urinary excretion of betaine was also significant in diabetic patients, which was associated with hyperglycemia and proximal tubular dysfunction^27^. Taken together, these suggested disturbed renal osmolyte regulation could lead to renal cell damage and deterioration of renal function. Increased urinary excretion of betaine and DMG in childhood NE was first reported in our study. We hypothesized that betaine and/or DMG might attribute to disturbed renal circadian rhythm, caused by impaired urinary concentration ability via osmolyte (tonicity)-dependent pathway.

Betaine has gained great attention as its anti-inflammatory or anti-oxidative functions on human disease in the recent days^36^. Considerable neuroscience re-search has shown the deficiencies of betaine and its precursor, choline, are associated with a variety of neurocognitive disorders (e.g., epilepsy, autism spectrum disorder, depression, schizophrenia and Alzheimer’s disease)^29,37,38^. And, systemic betaine supplementation was shown to benefit cognitive and memory functions in both animal and human studies^39^. The growing knowledge of betaine and other methyl group donors such as folate and vitamin B12 indicates their role as powerful epigenetic modulators essential for normal biological function and development. The deficiencies had a close linkage of modifications in histone and DNA methylation in brain^38,39^. Furthermore, the influence of osmolyte homeostasis on neural communication has been studied in many literatures^40–42^. Knight and his colleagues demonstrated a prominent role of brain betaine in neurotransmission^43^. They found betaine could provide neuroprotection via inhibitory neurotransmitter production and/or recycling, particularly involved in modulation of hippocampal functions. As NE and ADHD in children are widely accepted as the disorders of delayed brain development, our finding might speculate the role of betaine metabolism to play directly or indirectly in these two disorders.

Conclusively, in this pilot study, our results provided metabolomics insights into pathophysiological of childhood NE and its association with neurobehavioral disorders such as ADHD. We also suggested the likelihood of betaine in modulation of renal and central circadian clock systems in children with both NE and ADHD.

### The study limitations

There are several limitations and unclarified issues in our study. Firstly, the sufficient number of patient samples and the longer duration of follow-up will be required for better characterization and categorization of participants into different population subgroups. Although higher urinary expression of betaine was observed in the persistent bedwetting children with concomitant ADHD or anxiety, we do not analyze betaine concentration in blood and its interaction with other osmolytes or neuro-transmitters. Also, children diagnosed of neuropsychiatric disorders but without the problem of enuresis are not included in this study. Nearly half of the participants from the group of childhood NE collected their urine for metabolomics analysis before initiation of Minirin® treatment. Although our analysis showed very few metabolites significantly differentially expressed between NE children with and without receiving Minirin®, the potential of drug interference cannot be completely ignored. Therefore, further studies are needed for investigating the usefulness of urinary betaine as a non-invasive biomarker, and non-pharmacological approach for children with treatment-resistant enuresis affected by the presence of neurobehavioral disorders.

## Methods

### Participants and study design

This was a cross-sectional study consisting of 68 children while visits in outpatient clinics of Lin-Kou Chang Gung Memorial Hospital in Northern Taiwan from August 2017 to December 2018. Among the participants, 41 children were taken to see a pediatric nephrologist or urologist for bedwetting problems with and without daytime lower urinary symptoms such as incontinence, frequency or urgency. 27 children undergoing a well-child check were enrolled as health controls. A spot morning urine sample from individuals was collected during a morning visit. All of the participants had normal urinalysis and normal findings in renal ultrasonography. For 41 children with NE, furthermore, urine osmolality and the ratio of urine calcium to urine creatinine of spot morning urine samples were also measured, showing the values were within a normal range. At the time of urine collection, 21 bedwetting children did not start taking desmopressin acetate (Minirin®), but another 20 cases were already treated with Minirin® for one to three months. Nevertheless, desmopressin medication appeared not to be a factor for subsequent metabolomics analysis (Supplementary Table S2).

During the period of follow-up, bedwetting children who did not respond to the combination treatment consisting of Minirin® and oxybutynin or imipramine, and presented frequent NE (≥ three episodes per week) were referred to the psychiatric outpatient clinic in our hospital for evaluation. This study was approved by the local institutional review board (No. 201601179A3) of Lin-Kou CGMH, Taiwan. Written informed was obtained from children and/or their parent or legal guardian. All methods were carried out in accordance with the approved guidelines and regulations for medical research involving human subjects.

### ^1^H-NMR spectroscopy: urine sample preparation, data processing and analysis

Urine samples required for spectrum acquisition were prepared as described previously^44^. Firstly, 900 μL of urine was mixed with 100 μL of 1.5 M phosphate buffer in deuterium water containing 0.04% 3-(trimethylsilyl)-propionic-2,2,3,3-d4 ac-id sodium salt (TSP) as an internal chemical shift reference standard. The samples were vortexed for 20 s and centrifuged at 12000 g at 4 °C for 30 min. 600 μL supernatant was then transferred to a 5-mm NMR tube analysis. Sample spectra were measured by Bruker Avance 600 MHz NMR spectrometer (Bruker-Biospin GmbH, Karlsruhe, Germany). A total of 64 scans were collected for NMR spectra into 64 K computer data points with a spectral width of 10, 000 Hz (10 ppm). 1D NMR spectra were preprocessed by zerofills and exponential multiplication (0.3 Hz line broadening factor) prior to Fourier transformation. The acquired ^1^H-NMR spectra were then manually phased, baseline-corrected, and referenced the chemical shift to TSP (δ 0.0 ppm) using TopSpin 3.2 software (Bruker BioSpin, Rheinstetten, Germany).

Subsequently, the ^1^H-NMR spectra were imported into NMRProcFlow software, providing comprehensive tools for spectra processing, ppm calibration, baseline correction, alignment, spectra bucketing and data normalization^45^. Least-squares algorithm and parametric time warping were used to correct the misalignment spectra. Spectrum bucketing was performed using the method of intelligent bucketing and variable size bucketing^46^. Metabolites were identified using the Chenomx NMR Suite 8.1 software (Chenomx Inc., Edmonton AB, Canada). Urine spectra were specifically normalized to the integral of creatinine peak at δ 3.045 ppm to compensate the differences in urinary concentration. As established NMR data analysis in the previous experiment^44^, the normalized ^1^H-NMR spectra data were transformed using generalized log transformation (glog) and uploaded to MetaboAnalyst 4.0 (http://www.metaboanalyst.ca) to identify metabolites used for discrimination be-tween the groups using partial least squares-discriminant analysis (PLS-DA). Spectral variables were mean-centered and scaled using Pareto scaling. A further tenfold in-ternal cross-validation was performed to assess the quality of statistical models using the diagnostic measures R2 and Q2^47^. Metabolites with a variable importance in projection (VIP) score ≥ 1.0 or *p*-value < 0.05 were selected. The Kyoto Encyclopedia of Genes and Genomes database (KEGG) was employed to analyze the functional metabolic pathways.

### Statistical analysis

The baseline characteristics between bedwetting children with and without ADHD or anxiety disorder, and healthy controls were compared using Kruskai-Wallis test (GraphPad Prism 8.4.2). A *p*-value < 0.05 was considered statistically significance. The differences in metabolites between two groups were assessed using the Mann–Whitney test with the MetaboAnalyst web server. A false discovery rate (FDR) of 5% was applied to correct for multiple sets.

## Supplementary Information

Supplementary Table S1.

Supplementary Table S2.
