# Supplementary material for: Urine metabolic phenotyping in children with nocturnal enuresis and comorbid neurobehavioral disorders
Source: Sci Rep. 2021 Aug 16;11:16592. doi: 10.1038/s41598-021-96104-1 (PMC8368245; doi:10.1038/s41598-021-96104-1)
Supplement: Supplementary file 1 — Supplementary Table S1. [file 41598_2021_96104_MOESM1_ESM.pdf]

Table S1. PLS-DA parameters and permutation test for discrimination of global urinary metabolites between children with and without ADHD or anxiety disorder, and health controls (HC).

	Comp.	Q^2	R^2	Q^2/R^2	$P_{permutation}$
NE *vs*. HC	1	0.12	0.22	0.52	0.159
NE with coexisting ADHD/anxiety *vs*. HC	1	0.12	0.33	0.35	0.034
NE without ADHD/anxiety *vs*. HC	1	0.03	0.25	0.14	0.287
