# Supplementary material for: Urine metabolic phenotyping in children with nocturnal enuresis and comorbid neurobehavioral disorders
Source: Sci Rep. 2021 Aug 16;11:16592. doi: 10.1038/s41598-021-96104-1 (PMC8368245; doi:10.1038/s41598-021-96104-1)
Supplement: Supplementary file 2 — Supplementary Table S2. [file 41598_2021_96104_MOESM2_ESM.pdf]

TABLE S2. The VIP score and fold change of metabolites significantly differentially expressed between NE children with and without DDAVP medication at the time of urine sample collection.

		NE with vs. without DDAVP		
Metabolites	Chemical shift, ppm	VIP score*	Fold change†	*P*‡
Tyrosine	3.59-3.612	1.13	1.30	**0.024**
Glucose	4.634-4.674	1.17	1.29	**0.038**
Tiglylglycine	1.845-1.867	1.42	1.76	0.057
Succinate	2.402-2.418	1.34	0.66	0.067
N-Acetylglutamate	2.038-2.042	1.16	1.18	0.077
Glycine	3.569-3.577	1.24	1.25	0.079
Alanine	1.476-1.497	1.08	1.28	0.095
Fumarate	6.522-6.534	1.27	1.42	0.123
Valine	1.036-1.057	1.04	1.24	0.175
trans-Aconitate	6.588-6.606	0.97	1.22	0.180
Leucine	0.959-0.969	0.99	1.16	0.214
3-Hydroxyisobutyrate	1.064-1.09	0.98	1.44	0.217
Acetylsalicylate	2.338-2.358	1.62	0.70	0.223
Histidine	7.886-8.08	0.63	1.06	0.313
Lysine	1.696-1.769	1.49	1.17	0.386
Hypoxanthine	8.194-8.204	1.12	0.96	0.396
Taurine	3.419-3.447	1.11	0.97	0.472
Isoleucine	0.94-0.945	1.11	1.02	0.522
N,N-Dimethylglycine	2.911-2.938	1.01	1.04	0.550
3-Hydroxyisovalerate	1.27-1.277	0.72	1.11	0.610
N-Isovaleroylglycine	0.945-0.951	0.92	1.06	0.717
4-Hydroxyphenylacetate	6.85-6.881	0.53	0.95	0.832
Methanol	3.352-3.399	1.12	0.73	0.861
Creatine phosphate	3.953-3.964	0.72	0.99	0.889
N-Acetylglutamine	2.042-2.046	0.55	0.98	0.915

*VIP score were obtained from PLS-DA. †Fold changes were calculated by dividing the value of metabolites in NE children with by without DDAVP medication. ‡All *p*- values < 0.05, which is in bold, are significant. VIP, Variable Importance in Projection. NE, nocturnal enuresis; DDAVP, desmopressin (Minirin®).
